# Integrative pharmacovigilance and AI-based framework uncovers potential drug triggers in juvenile idiopathic arthritis

**DOI:** 10.3389/fimmu.2025.1653003

**Published:** 2025-11-03

**Authors:** Qiang Luo, Yuxiao Chen, Dawei Liu, Xinlin Wu, Jun Yang, Haiguo Yu, Hongmei Song, Junfeng Wu, Jingyi Zhao, Xuemei Tang

**Affiliations:** ^1^ Department of Rheumatology and Immunology, Children’s Hospital of Chongqing Medical University, Chongqing Key Laboratory of Child Rare Diseases in Infection and Immunity, National Clinical Research Center for Child Health and Disorders, Ministry of Education Key Laboratory of Child Development and Disorders, Children's Hospital of Chongqing Medical University, Chongqing, China; ^2^ Rheumatology and Immunology Department of Shenzhen Children's Hospital, Shenzhen, China; ^3^ Department of Rheumatology and Immunology, Nanjing Children's Hospital, Nanjing, China; ^4^ Pediatric Department of Peking Union Medical College Hospital, Chinese Academy of Medical Sciences, Beijing, China; ^5^ Institute of Traditional Chinese Medicine, Chengde Medical University, Chengde, China

**Keywords:** juvenile idiopathic arthritis, machine learning models, FAERS, toxicogenomic, pharmacovigilance, systems immunology, multicenter study

## Abstract

**Background:**

Management of juvenile idiopathic arthritis (JIA) relies heavily on long-term pharmacotherapy, yet an increasing number of case reports suggest that some drugs may themselves precipitate or worsen the disease. But systematic methods for detecting these safety signals in pediatric cohorts are still lacking.

**Methods:**

We screened 10,012,438 reports from the FAERS database using four disproportionality algorithms (ROR, PRR, EBGM, and BCPNN) to identify potential drug and JIA associations. Three complementary machine learning models were developed, including DMPNN, GCN, and SVM, trained on molecular descriptors, chemical fingerprints, and structural graphs to stratify high-risk compounds. Toxicogenomic profiles were generated using ProTox-3.0, and drug–disease target overlap and pathway enrichment were assessed using the CTD and GeneCards databases. External validation relied on our own newly generated transcriptomic data: (i) our newly generated bulk RNA-seq dataset from 47 individuals (39 JIA patients and 8 controls) and (ii) a multi-center single-cell RNA-seq compendium that combined 21 in-house PBMC profiles obtained at four Chinese pediatric hospitals with 9 publicly available systemic juvenile idiopathic arthritis (sJIA) samples. Two of the in-house sJIA patients were sampled longitudinally, before and one month after IL-6-receptor-inhibitor therapy permitting assessment of treatment-induced transcriptomic shifts. Drug-signature activity was quantified with single-sample GSEA for the bulk data and AddModuleScore for the single-cell data.

**Results:**

We identified drugs with consistent positive signals across all four FAERS-based disproportionality algorithms. Machine learning models (DMPNN, GCN, SVM) independently confirmed 23 high-risk compounds, with 22 overlapping across all models and predicted risk scores >0.60. Among these, lansoprazole and aripiprazole showed strong signals in both pharmacovigilance and DMPNN predictions. Further toxicogenomic analysis revealed immune toxicity patterns overlapping with JIA-related gene targets and pathways. Notably, bulk RNA-seq and single-cell RNA-seq validation demonstrated that lansoprazole signatures were significantly enriched in monocyte from sJIA patients. This multi-level convergence supports the hypothesis that certain non-antirheumatic drugs may aggravate JIA-like inflammation, particularly within the systemic subtype.

**Conclusions:**

In this study, we identify lansoprazole as a likely instigator of systemic juvenile idiopathic arthritis, underscoring that proton-pump inhibitors should be used judiciously in children at autoimmune risk and providing a generalizable playbook for rare-disease pharmacovigilance.

## Introduction

Medication use in children with Juvenile Idiopathic Arthritis (JIA) is widespread, with most patients requiring long-term pharmacologic therapy involving immunosuppressants, nonsteroidal anti-inflammatory drugs (NSAIDs), glucocorticoids, and biologic agents (1–3). While these medications are essential for disease control, growing attention has been drawn to the possibility that certain drugs may themselves act as environmental triggers for the onset or exacerbation of JIA in genetically or immunologically predisposed individuals (4, 5).

Evidence from pharmacovigilance systems and published literature has implicated a wide range of drug classes, including vaccines, biologics, antimicrobials, and neuropsychiatric agents, in the development of idiopathic arthritis (IA), including JIA (6–8). These effects may be mediated through mechanisms such as molecular mimicry, immune activation, and gut microbiota disruption. Among vaccines, the rubella component of MMR is well known to cause transient arthritis in patients (9), and case reports have linked immune-mediated arthritic reactions to hepatitis B and COVID-19 vaccines (8, 10). Biologic agents such as TNF-α inhibitors and immune checkpoint inhibitors have also been associated with paradoxical inflammatory arthritis, supported by safety signals from the FAERS and VigiBase databases (11–15). Additional suspected triggers include interferon-α, repeated early-life exposure to broad-spectrum antibiotics, and certain psychotropic agents (13, 16, 17). However, the precise causal relationships between these pharmacological exposures and JIA remain to be fully elucidated.

However, existing evidence remains fragmented, often derived from isolated case reports or underpowered observational studies. And, preclinical models frequently fail to capture pediatric-specific immune dynamics, and real-world ADR data in children with JIA are sparse and delayed due to disease rarity and underreporting. These limitations underscore the urgent need for comprehensive, data-driven approaches to systematically identify and evaluate drugs that may not only treat but also induce or worsen JIA. Artificial intelligence-driven frameworks are increasingly being applied across diverse biomedical fields. In arthritis and immune-mediated diseases, recent advances demonstrate their promise as systematic tools for integrating pharmacovigilance, multi-omics, and clinical datasets to improve the assessment of drug safety and therapeutic efficacy (18, 19).

In this study, we extracted JIA-related ADR reports from the U.S. Food and Drug Administration’s Adverse Event Reporting System (FAERS), explored multiple data preprocessing strategies, and compared three classification approaches for stratifying high-risk versus low-risk drugs. Predictive models were developed using various drug representation techniques—including 2D molecular descriptors, molecular fingerprints (ECFP4 and MACCS), and molecular graphs—and were trained using both classical machine learning algorithms and deep learning architectures. To enable external validation, we also leveraged data from an independent cohort of JIA patients from 5 centers. Drug-specific gene signature enrichment scores were computed to further support the model’s predictive capacity. This AI-driven framework provides a valuable tool for systematically identifying medications with the potential to induce JIA, thereby enhancing drug safety assessments and guiding informed decision-making in pediatric rheumatology and pharmacovigilance.

## Methods

**Figure 1:**
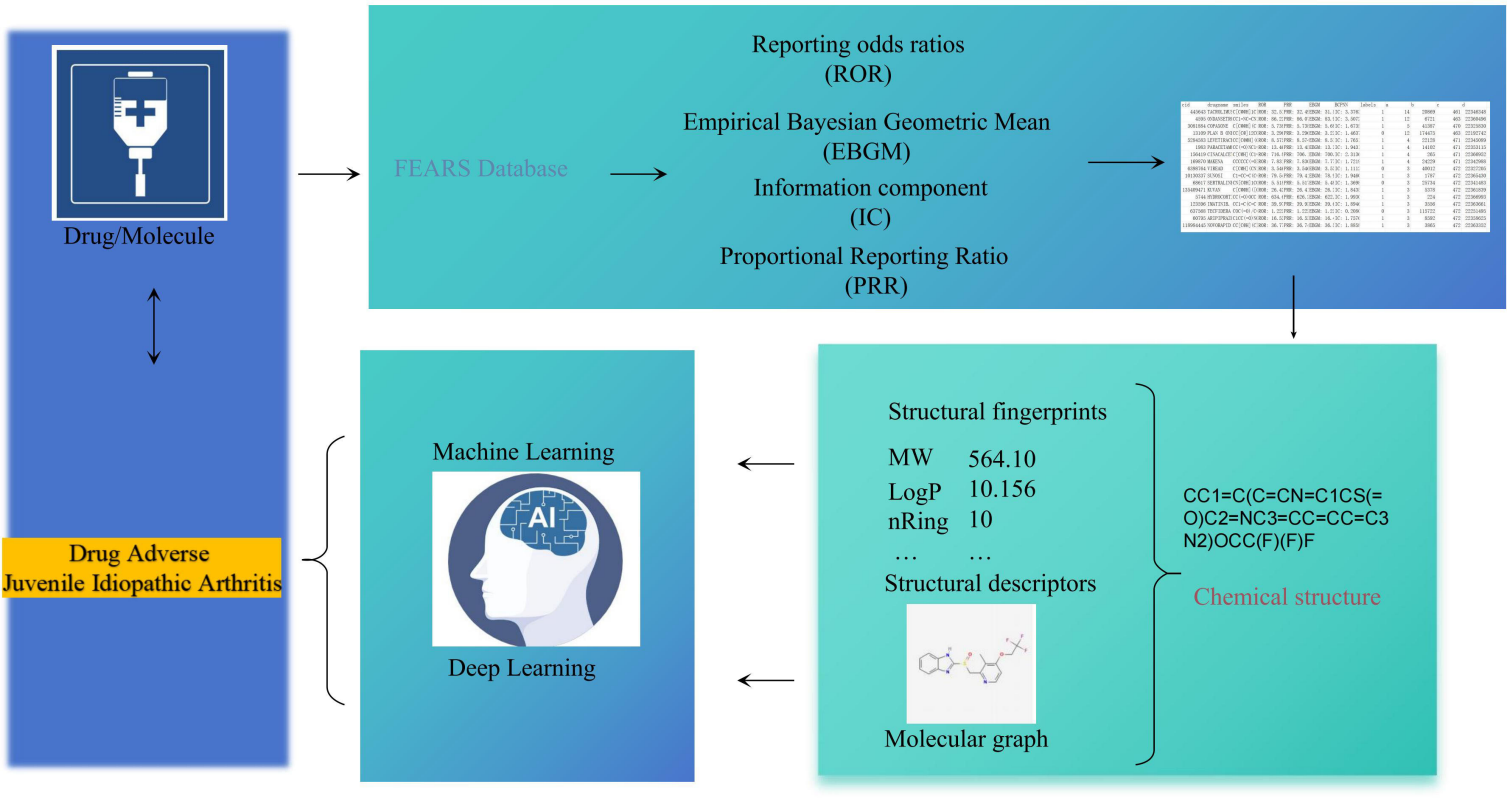
The technology roadmap is included in this study.

The process of constructing an AI-based predictive model for the identification of JIA encompasses multiple essential stages, including data acquisition and preprocessing, computational analysis of molecular features, model development. A schematic overview of these procedures is provided in **Figure 1**.

### Data acquisition

Data analyzed in this study were collected from the FAERS database (https://fis.fda.gov/extensions/FPD-QDE-FAERS/FPD-QDE-FAERS.html), where adverse drug reactions (ADRs) are systematically categorized according to the MedDRA standard terminology (20). To enable a comprehensive assessment, all adverse event reports pertaining to JIA were consolidated. As part of the FAERS data preprocessing, duplicate entries were removed, and only the latest records were retained, yielding a dataset comprising 10012438 adverse event reports recorded between January 2004 and December 2024. The associated drugs were determined, and their generic names were retrieved from the PubChem database (https://pubchem.ncbi.nlm.nih.gov/). Various analytical strategies were applied to distinguish drugs associated with higher versus lower risk for JIA-related adverse reactions across different hierarchical levels.

All compounds included in the dataset were restricted to small-molecule drugs, with their molecular structures retrieved from the PubChem database and represented as Simplified Molecular Input Line Entry System (SMILES) strings. Drugs lacking available SMILES information were excluded from further analysis. To maintain structural consistency and enhance accuracy, all molecular structures were standardized using the “wash” protocol implemented in the Molecular Operating Environment (MOE) software (version 2022.02, Chemical Computing Group, Montreal, QC, Canada). Standardization procedures involved the removal of salts and minor components, deprotonation of strong acids and bases, and the addition of explicit hydrogen atoms, thereby facilitating the calculation of molecular descriptors and the construction of molecular graphs.

External validation relied on our own newly generated transcriptomic data: For bulk RNA-seq, PBMCs were collected from 39 patients with non-systemic juvenile idiopathic arthritis (non-sJIA), 16 patients with systemic juvenile idiopathic arthritis (sJIA), and 8 healthy controls conducted at the Children’s Hospital of Chongqing Medical University. All patients enrolled in the study were diagnosed based on the classification criteria established by the International League of Associations for Rheumatology (ILAR) (21).

For single-cell RNA sequencing (scRNA-seq), PBMCs were prospectively collected from 27 patients with sJIA across five centers and 6 healthy controls. In addition, paired PBMC samples were obtained from 2 sJIA patients before and one month after IL-6 inhibitor treatment. Clinical samples obtained from the following institutions: Children’s Hospital of Chongqing Medical University (10 sJIA), Peking Union Medical College Hospital (1 sJIA), Children’s Hospital of Nanjing Medical University (3 sJIA), Shenzhen Children’s Hospital (4 sJIA), and Cincinnati Children’s Hospital Medical Center (9 sJIA) (GSE207633). All patients enrolled in the study were diagnosed based on the classification criteria established by the ILAR (21).

### Methods for classifying drugs into high-risk and low-risk categories

Signal detection of ADRs within spontaneous reporting systems commonly relies on Bayesian-based approaches, such as the BCPNN, and frequency-based metrics, including the ROR, PRR and EBGM. In the present study, four analytical methods were utilized to evaluate the risk of JIA-associated ADRs across different drugs (22).

### Time-to-onset

The time-to-onset (TTO) of ADE is analyzed, TTO is defined as the time period between the date the ADE occurred (EVENT_DT in the DEMO file) and the date the medication started (START_DT in the THER file). To ensure accuracy and reliability, any inaccurate date entries, missing data, or input errors (such as instances where EVENT_DT appears before START_DT) are removed from the analysis. The median, interquartile distance (IQR), and Weibull shape parameter (WSP) are utilized to evaluate the TTO (22, 23). The Weibull distribution is a statistical model that characterizes the shape of failure or event time data. It is defined by two parameters: scale (α) and shape (β). The Weibull shape parameter (β) is particularly relevant for evaluating TTO patterns. Different values of β correspond to different failure types: Early failure types: β < 1, with a 95% confidence interval (CI) also less than 1. These types exhibit a diminishing ADE risk over time, indicating that the occurrence of ADE decreases as time progresses. Random failure types: β equals or approximates 1, with its 95% CI encompassing the value 1. These types entail a consistent ADE hazard rate over time, suggesting a relatively stable risk of ADE occurrence. Wear failure types: β > 1%, with a 95% CI also greater than 1. These types indicate an escalating ADE risk as time progresses, meaning that the likelihood of experiencing an ADE increases over time. In simulations of Weibull distribution, the Kolmogorov-Smirnov (KS) test, which involves D statistics and P-value, is a widely used technique to assess the alignment between simulation results and theoretical or observed distributions.

### Molecular structure characterization

In traditional machine learning (ML) frameworks, molecular structures are typically represented through molecular descriptors and fingerprints, whereas deep learning (DL) approaches employ molecular graphs. In the present study, drug structures were characterized using a combination of 2D molecular descriptors, two distinct types of fingerprints (ECFP4 and MACCS), and molecular graphs.

### 2D molecular descriptors

A total of 83 two-dimensional (2D) molecular descriptors were computed using the rdkit.Chem.Descriptors function from the RDKit toolkit (version 2023.9.6). This descriptor set includes features such as molecular weight, partition coefficient (log P, calculated via Wildman and Crippen methods), the number of hydrogen bond donors and acceptors (NumHDonors and NumHAcceptors), topological parameters (TPSA, kappa indices 1–3, BertzCT), compositional attributes (NumRings, NumAromaticRings), and electrotopological state indices (Estate). The complete list of calculated descriptors is available in the RDKit documentation (https://www.rdkit.org/docs/GettingStartedInPython.html#list-of-available-descriptors) and is collectively referred to as Des in this study.

### Fingerprints

The MACCS fingerprint encodes the presence (1) or absence (0) of predefined chemical features within a 166-bit binary vector. In contrast, the Extended Connectivity Fingerprint (ECFP4) addresses molecular isomorphism by decomposing molecules into circular fragments and encoding each atom based on its environment. The ECFP4 fingerprint, characterized by a radius of 2, is typically represented as a 1024-bit binary vector. Both MACCS and ECFP4 fingerprints were generated using the RDKit package (version 2023.9.6).

### Molecular graphs

Graph-based molecular representation methods apply convolutional operations to capture structural features. In this framework, a molecule is abstracted as a graph G = (V, E), where nodes (V) correspond to atoms, each associated with a feature vector (X_v_), and edges (E) represent bonds with feature vectors (E_km_), denoting connections between atoms k and m. Thus, molecules are modeled as interconnected nodes and edges. Deep learning (DL) techniques leverage convolutional transformations over these graphs to learn latent molecular representations, which are subsequently utilized during the readout phase to predict various molecular properties.

### Model construction

The evaluation of model performance for JIA-associated adverse drug reactions (ADRs) encompassed both traditional machine learning (ML) and deep learning (DL) strategies. Descriptor-based models were generated utilizing one ML algorithms: support vector machines (SVM). Concurrently, deep learning frameworks were established based on two graph neural network (GNN) architectures: the graph convolutional network (GCN) and the directed message passing neural network (DMPNN).

### Descriptor-based models

Descriptor-based models were established using two types of molecular fingerprints, ECFP4 and MACCS, along with a set of 2D molecular descriptors generated via RDKit. For the support vector machine (SVM) model, a radial basis function (RBF) kernel was applied.

### Graph convolutional network model

The GCN model applied a scalable semi-supervised learning strategy designed for graph-structured datasets. It leveraged a localized first-order approximation of spectral graph convolutions to enable direct operations on graph representations. Hyperparameters were optimized during model construction, including the number of hidden units per GCN layer, the use of residual connections (enabled), application of batch normalization (enabled), dropout rate, hidden dimensions for the multilayer perceptron (MLP) predictor, and predictor dropout rate.

### Directed message passing neural network model

The DMPNN model employed bond-centered convolutional operations to encode molecular structures, thereby avoiding unnecessary cyclic message propagation. Previous studies have indicated that combining DMPNN with external molecular features improves model performance, as 2D molecular descriptors provide global contextual information while DMPNN captures local structural features. In this study, DMPNN was enhanced through the integration of additional feature vectors at the molecular level, resulting in two variants: DMPNN-Des (with 2D molecular descriptors) and DMPNN-ECFP4 (with ECFP4 fingerprints). Hyperparameter optimization was conducted following the Chemprop 2.0.4 framework, targeting variables such as the depth of the message passing phase, hidden dimension in the encoder, number of layers in the feedforward neural network module, batch size, activation function for encoding layers, and dropout probability within the encoder.

The descriptor-based models (SVM) was implemented using the scikit-learn library (version 0.20.1) in a Python 3.8 environment. Graph-based models, including the GCN and the GNN, were developed with the Deep Graph Library (DGL, version 0.4.1) using PyTorch as the backend. The directed message passing neural network (DMPNN) was constructed employing the Chemprop package (version 1.0). All deep learning models were trained using the Adam optimizer, and hyperparameters were optimized systematically through Bayesian optimization techniques.

### Model evaluation

The dataset was randomly partitioned into training, validation, and test subsets in an 8:1:1 ratio. To assess model robustness and generalization ability, a 5-fold cross-validation strategy was employed during training. Specifically, the dataset was divided into five equal parts, with four parts used for model training and the remaining part for validation in each iteration, cycling through all five partitions. Final model performance was evaluated using the independent test set. Model discrimination ability was primarily assessed by calculating the area under the receiver operating characteristic curve (AUC), which reflects the overall capability of the model to distinguish between high-risk and low-risk drugs.

### Analysis of substructure alerts

To investigate the relationship between molecular substructures and JIA-associated ADRs, we employed the fears toolkit developed by our research group. For each molecule, the model-derived predictive outcome was used to assess the contribution of its corresponding substructures to ADR risk, enabling a systematic and data-driven identification of potential substructure alerts.

### FAERS sensitivity analysis for co-medications

Concomitant use of multiple medications is common in clinical practice and may influence the occurrence of JIA, thereby affecting signal detection. To enhance the robustness of our analysis and minimize potential confounding bias, we performed a sensitivity analysis by excluding reports that involved concomitant therapies, thereby improving the reliability of AE signal detection.

### Toxicity prediction

To assess the potential toxicity of the investigated compounds, we employed the ProTox-3.0 webserver (https://tox.charite.de/protox3/), a publicly available platform for in silico toxicity prediction. ProTox-3.0 integrates molecular similarity assessment, fragment-based analysis, and machine learning algorithms to predict 61 toxicity endpoints, including acute toxicity, organ-specific toxicity, molecular initiating events, toxicity pathways, metabolism, and toxicity targets. Canonical SMILES (Simplified Molecular Input Line Entry System) strings were submitted for each compound via the ProTox-3.0 interface. Toxicity profiles were predicted based on structural similarity to known toxicants and model-derived inference. Outputs included predicted toxicity classes, LD_50_ values, and associated confidence scores for each endpoint. The platform also provided visualization tools such as toxicity radar plots and interaction network diagrams to aid interpretation. All predictions were performed using the default configuration settings of the ProTox-3.0 platform.

### Identification of potential mechanistic targets via intersection analysis

To elucidate the potential mechanistic basis of drugs in the context of JIA, we performed a target intersection analysis between drug-associated targets and disease-related genes. First, the molecular targets of drugs were retrieved from the CTD database (https://ctdbase.org/). Next, JIA genes were identified using GeneCards (https://www.genecards.org/) by querying the term “Juvenile Idiopathic Arthritis”. All genes with a GeneCards relevance score ≥ 10 were retained as putative disease-related targets. The intersection of the two gene sets—i.e., shared targets between drugs and JIA was computed using custom scripts in R. The overlapping targets were considered potential mechanistic mediators and were subjected to further functional enrichment and pathway analysis.

### ssGSEA-based drug signature scoring and association with JIA subtypes

To quantify the transcriptional activity of drug-specific gene signatures across patients, we conducted single-sample gene set enrichment analysis (ssGSEA) using the GSVA packagein R. The “limma” R package was used to remove the batch effect and eliminate the sample sets with excessive differences (24). Drug-associated gene sets were curated from [insert source, e.g., LINCS, DrugMatrix, or previously published datasets], representing transcriptional responses to a range of pharmacologic compounds.

For each patient, ssGSEA generated an enrichment score reflecting the coordinated up- or downregulation of genes within each drug signature. These scores served as proxies for drug-specific transcriptional activation at the individual level.

To investigate the relationship between drug signature activity and clinical heterogeneity, we compared ssGSEA enrichment scores across major JIA subtypes. Statistical comparisons were performed using one-way ANOVA tests. Subtype-specific enrichment patterns were visualized via boxplots to identify compound signatures preferentially associated with distinct JIA phenotypes.

### Data processing and cell clustering of individual cases

Preprocessed gene expression matrices from each sample were independently analyzed using RStudio (v4.0.2) and the Seurat package (v4.1.0). Initial quality control excluded ribosomal genes, genes expressed in fewer than three cells, and cells expressing fewer than 200 genes. And cells with >16% mitochondrial gene content, <3% ribosomal gene content, or >0% hemoglobin gene content were considered low quality and excluded from further analysis (25).

Monocytes were identified based on high expression of LYZ, FCN1, AIF1, and S100A12; NK cells by GNLY, KLRD1, NKG7, KLRB1, and KLRK1; B cells by CD79A, MS4A1, MZB1, and JCHAIN; megakaryocytes by ITGA2B and GP9; dendritic cells by TMPO and GIMAP4; and T cells by CD3E, TCF7, RACK1, IL7R, and IFITM1. Cluster identities were inferred based on the expression of characteristic markers.

### Data integration with batch effect collection

To normalize and integrate multi-sample scRNA-seq datasets, we applied SCTransform-based normalization followed by Harmony batch correction. Specifically, each dataset was normalized using SCTransform with regularized negative binomial regression, and the top variable features were used for PCA reduction (26). Batch effects across samples (denoted by orig.ident) were corrected using the Harmony algorithm, yielding a shared low-dimensional representation. Following Harmony integration, we performed UMAP (RunUMAP (reduction = “harmony”)) (27). Graph-based clustering was conducted using the FindNeighbors and FindClusters functions based on the first 20 Harmony-corrected principal components. To determine the optimal clustering resolution, we tested a range of resolutions (0.1–1.0) and evaluated the clustering structure using Clustree. Based on visual inspection of UMAP plots and cluster tree topology, we selected resolution = 0.9 for downstream cell population definition. Clusters with low unique feature counts or high mitochondrial content were excluded as low-quality or apoptotic cells. All visualizations were generated using DimPlot.

### Scoring and comparison of gene set activity across single-cell populations

To assess the drug-specific gene at single-cell resolution, we employed the AddModuleScore function in the Seurat package in R. This method calculates a relative expression score for each cell by averaging the expression of genes within the target set and adjusting for background expression using control gene sets matched by average expression. Statistical comparisons of gene set activity across annotated cell populations were performed using Wilcoxon rank-sum tests. Visualization was carried out using functions from the Seurat and ggplot2 packages.

### Cell-based experimental validation

Human THP-1 monocytes were maintained in RPMI-1640 medium supplemented with 10% fetal bovine serum (FBS) and 1% penicillin–streptomycin at 37°C in a 5% CO_2_ incubator. Differentiation into macrophages was induced by exposure to phorbol 12-myristate 13-acetate (PMA, 100 ng/mL) for 24h. To evaluate cytotoxicity, cells were seeded into 96-well plates (1×10^4^ cells/well) and treatedgs with potential candidate drugs at 0, 10, 20, 40, or 80 μM for 24h, followed by CCK-8 measurement of cell viability at 450 nm. For lysosomal activity, differentiated THP-1 macrophages (1×10^6^ cells/dish) were exposed to increasing concentrations of lansoprazole (0–80 μM, 24h), stained with LysoTracker Green and DAPI, and imaged by laser confocal microscopy. To assess inflammasome activation, cells were first primed with lipopolysaccharide (LPS, 1 μg/mL, 3h) to induce baseline expression of pro-IL-1β and NLRP3, after which lansoprazole (0–80 μM, 24h) was applied; supernatants were collected and IL-1β and IL-18 levels quantified by ELISA according to manufacturer instructions.

### Ethical considerations

Ethical clearance was obtained from the Institutional Review Board of the Children’s Hospital of Chongqing Medical University (Approval No. (2023)IRB(STUDY) No.351). As a retrospective analysis based solely on existing medical records from prior clinical visits, the study posed no additional risk to the participants.

## Results

### Descriptive results of pharmacovigilance analysis

**Figure 2:**
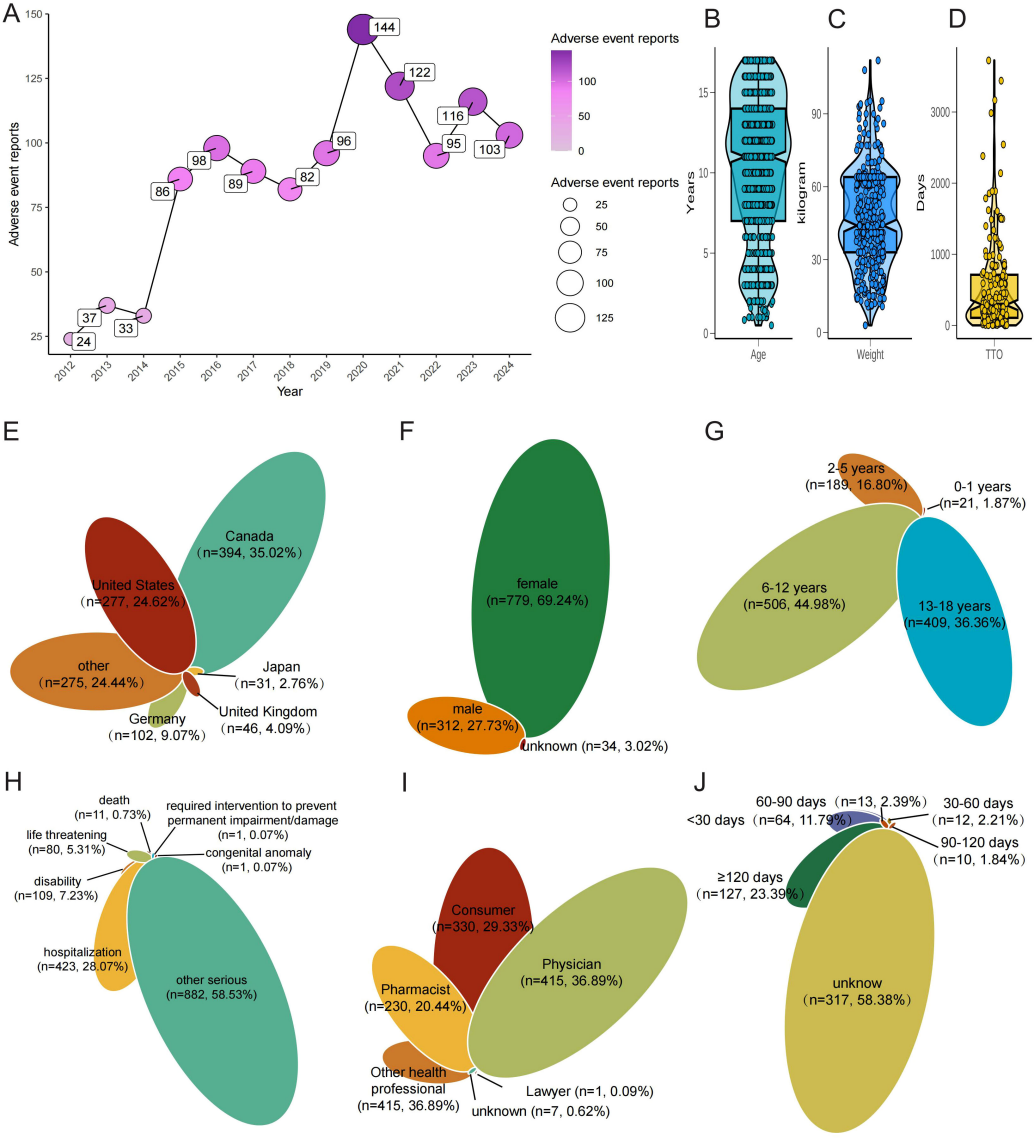
Baseline clinical characteristics of FAERS reports involving JIA. **(A)** Annual number of JIA-related reports (2004–2024), with a surge in 2020. **(B)** Age distribution of patients (violin plot). **(C)** Weight distribution of patients (violin plot). **(D)** Time-to-onset (TTO) from drug initiation to first JIA report (boxplot, Weibull β inset). **(E)** Country of origin of reports (top 10 + Other). **(F)** Sex distribution. **(G)** Age sub-groups (0–2, 3–5, 6–11, 12–17 years). **(H)** Clinical outcomes (Hospitalisation, Disability, Life-threatening, Death, Other). **(I)** Reporter roles (Physician, Consumer, Pharmacist, Other). **(J)** TTO grouped as ≤30 d, 31–180 d, >180d.

A total of 1125 ADE associated with JIA were documented in the FAERS database from Q1–2004 to Q4 2023 (**Figure 2**). Notably, the incidence of reported JIA peaked in 2020, accounting for 144 cases (**Figure 2A**). Subsequently, there was a decline in reports starting from 2020, although an overall upward trend persisted throughout the specified period. Examining the chronological pattern of ADR reports, peak activity for JIA cases was observed in the years 2020, 2021, 2023 and 2024. And, The majority of patients mentioned in ADE reports were over the age of 6-12 (**Figures 2B, G**). Patient weights ranged from 5kg to nearly 100kg, with a median of ~38 kg and an inter-quartile range of roughly 28–60 kg. The violin plot reveals two density peaks, one around 30–35 kg (younger school-age children) and another near 55–65 kg (older adolescents), mirroring the bimodal age structure of the cohort (**Figure 2C**). **Figure 2D** reveals a markedly right-skewed TTO distribution, and half of the reported reactions occurred within roughly the first four months of drug exposure (**Figures 2D, J**) Geographically, Canada (35.02%) and the United States (24.62%) contributed the largest shares, followed by Germany, the United Kingdom and Japan (**Figure 2E**). Gender distribution analysis revealed that among the reported cases excluding those lacking gender details, females accounted for 69.24% of the cases, while males represented 27.73% (**Figure 2F**). Concurrently, out of 1125 ADR reports involving hospitalization occurred in approximately 23.60% of cases, deaths were recorded at a rate of 0.73%, and disability were rare, evident in 7.32% of cases (**Figure 2H**). Physicians submitted the plurality of cases (36.89%), while consumers and pharmacists accounted for 29.33% and 20.44%, respectively; contributions from other health professionals, lawyers and unknown reporters were minor (**Figure 2I**).

### Data mining on a JIA-inducing drug

Signal detection analysis identified a total of 40 drug entities with consistent positive associations with JIA across all four disproportionality algorithms (ROR, PRR, EBGM, and BCPNN). Notably, ciclosporin, aripiprazole, fluoxetine hydrochloride, lorazepam, clobazam, duloxetine, gabapentin, methadose, cetirizine, cetirizine hydrochloride, and lansoprazole showed strong and recurrent signals, indicating a close association with JIA risk (**Supplementary Table S1**).

### AI-based risk prediction

To evaluate compound-level risk prediction for JIA, we compared three distinct modeling approaches: a directed message passing neural network (DMPNN), a graph convolutional network (GCN), and a support vector machine (SVM). All three models demonstrated strong discriminatory performance and collectively identified a set of drugs potentially associated with the risk of JIA.

**Figure 3:**
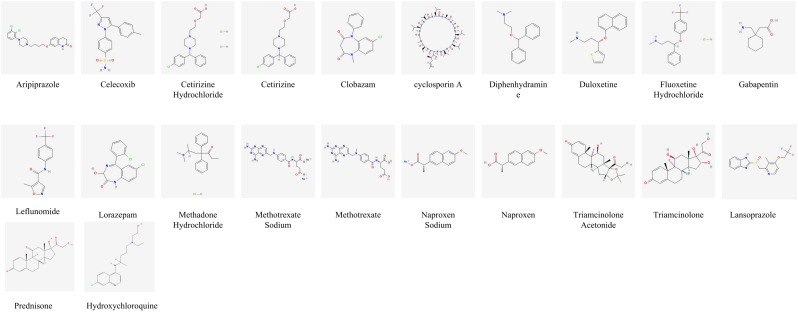
Risk prediction based on artificial intelligence and molecular formula of drugs related to JIA.

**Figure 4:**
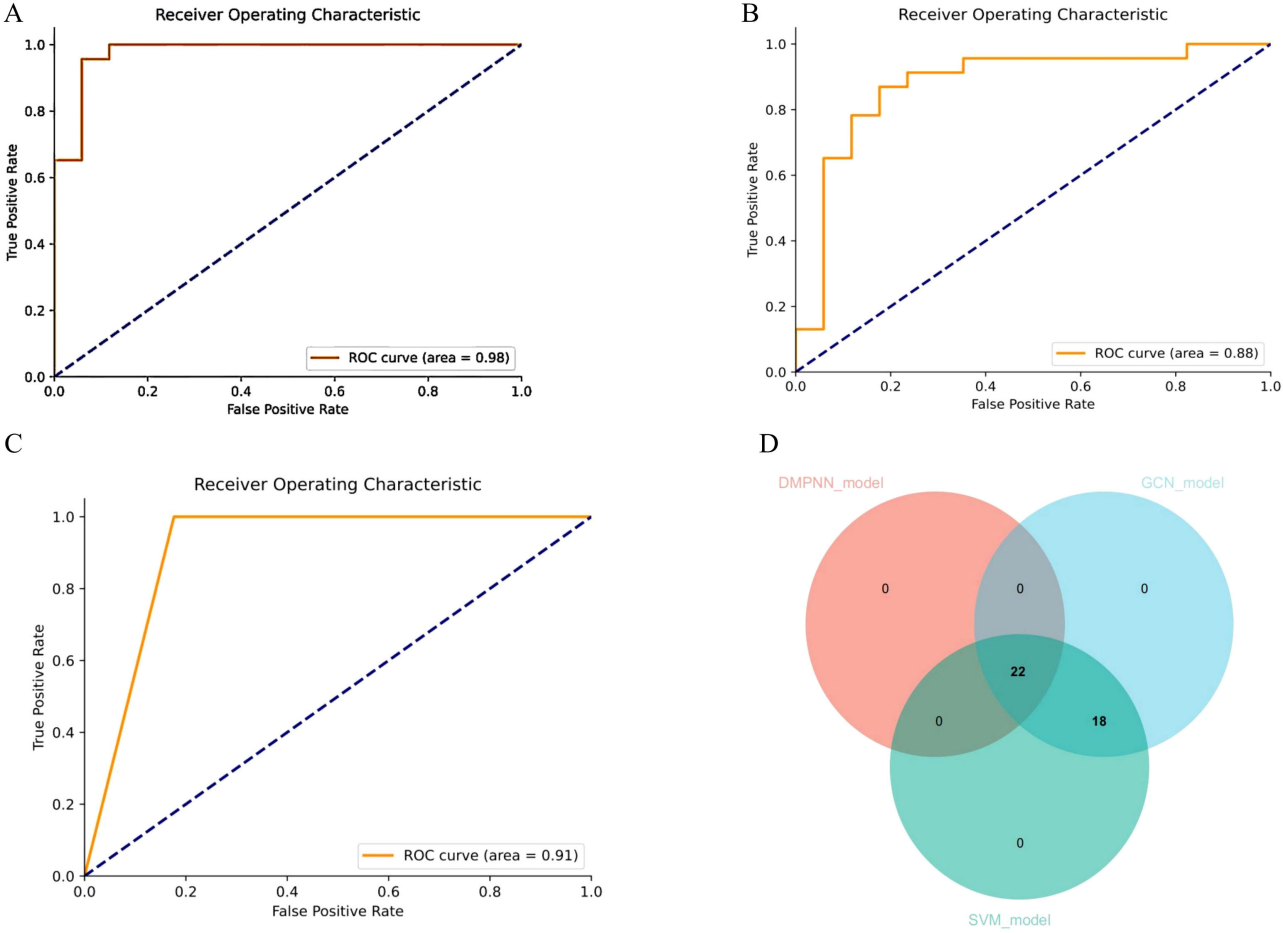
Receiver operating characteristic curve (ROC) demonstrating the predictive performance of different models for drug-associated JIA risk. **(A)** ROC curve of the DMPNN model. **(B)** ROC curve of the GCN model. **(C)** ROC curve of the SVM model. **(D)** Overlap of drug predictions (Venn diagram).

The DMPNN model generated compound-level risk scores for 22 candidate drugs potentially associated with JIA (**Supplementary Table S2**, **Figure 3**), with excellent performance supported by a high area under the receiver operating characteristic curve (AUC=0.98) (**Figure 4A**). Similarly, the GCN model identified the same set of 22 high-risk compounds (**Supplementary Table S3**), achieving a predictive performance of AUC=0.88 (**Figure 4B**). The SVM model also yielded consistent results, detecting 23 compounds closely linked to JIA occurrence (**Supplementary Table S4**), with an AUC of 0.91 (**Figure 4C**), further confirming the robustness and agreement across modeling approaches.

Subsequent intersection of the outputs from the three models revealed a shared subset of 22 overlapping drugs, each consistently predicted to be associated with JIA. Notably, all 22 drugs received predicted risk scores above 0.60 across all three models (**Figure 4D**, **Table 1**), underscoring their strong prioritization and potential clinical relevance in the context of JIA-related adverse drug events.

**Table 1:** AI-Based risk prediction.

cid	Drugname	Labels	DMPNN_pred	GCN_pred	SVM_pred
3883	LANSPORAZOLE	1	0.6304	0.9691	1
126941	METHOTREXATE|METHOTREXATE.|METHOTREXATE (TRADE NAME UNKNOWN)	1	0.7057	0.9809	1
31307	TRIAMCINOLONE	1	0.7293	0.9849	1
5284373	CICLOSPORIN	1	0.7445	0.9886	1
60795	ARIPIPRAZOLE|ARIPIPRAZOLE.	1	0.7541	0.9599	1
3446	GABAPENTIN	1	0.8049	0.9878	1
2662	CELECOXIB.|CELECOXIB	1	0.8107	0.9936	1
156391	NAPROXEN.|NAPROXEN	1	0.8202	0.908	1
3899	LEFLUNOMIDE.|LEFLUNOMIDE	1	0.8328	0.9871	1
6436	TRIAMCINOLONE ACETONIDE.|TRIAMCINOLONE ACETONIDE	1	0.8481	0.9893	1
3958	LORAZEPAM	1	0.8713	0.9724	1
11329481	METHOTREXATE SODIUM.|METHOTREXATE SODIUM	1	0.8752	0.9976	1
62857	FLUOXETINE HYDROCHLORIDE	1	0.9193	0.9875	1
23681059	NAPROXEN SODIUM ({= 220 MG)|NAPROXEN SODIUM	1	0.9301	0.9871	1
2678	CETIRIZINE	1	0.9376	0.9678	1
55182	CETIRIZINE HYDROCHLORIDE	1	0.9423	0.9675	1
60835	DULOXETINE.	1	0.9584	0.9007	1
2789	CLOBAZAM	1	0.96	0.9534	1
14184	METHADOSE	1	0.9762	0.9791	1
3100	DIPHENHYDRAMINE	1	0.9861	0.977	1
5865	PREDNISONE.	1	0.6362	0.9457	1
3652	HYDROXYCHLOROQUINE	1	0.6249	0.9778	1

To further characterize the 22 high-confidence drugs identified by all three models, we conducted a comprehensive review of the existing literature and official prescribing information. We found that only a small subset, most notably aripiprazole and lansoprazole have previously been reported in association with joint-related adverse events (**Table 2**). Aripiprazole has been linked to arthralgia and joint stiffness in post-marketing surveillance, while lansoprazole has been implicated in drug-induced lupus erythematosus (DILE), which may present with arthritis-like symptoms. For the remaining drugs, no published evidence or product labeling currently supports a role in exacerbating arthritis or triggering systemic inflammatory responses resembling JIA. These findings highlight the potential of our predictive framework to uncover previously unrecognized safety signals and reinforce the need for further pharmacological and mechanistic investigation.

**Table 2:** Review of literature and drug instructions for aripiprazole and lansoprazole.

Drug name	Used for treatment treat JIA	Reported Inducing/increase JIA?	Main sources of evidence	Cite
Aripiprazole	NO	MAY	Post-marketing surveillance has identified arthralgia (1–10%) and joint stiffness (0.1–1%) as uncommon adverse events. Although arthritis-like symptoms have been observed, the exact pathogenic mechanism remains unclear.	based on drug side effect data (https://www.drugs.com/sfx/abilify-side-effects.html#:~:text=,syndrome%2C%20sciatica%2C%20skeletal%20injury%2C%20stiffness)
Lansoprazole	NO	MAY	Lansoprazole itself is not an antirheumatic agent, but proton-pump inhibitors (PPIs) have been reported to induce drug-induced lupus erythematosus (DILE), a condition that can manifest with fever, arthralgia, and even frank arthritis. Current hypotheses suggest a loss of immune tolerance in genetically susceptible individuals, yet the precise mechanism is still unknown.	based on drug side effect data (https://www.mayoclinic.org/drugs-supplements/lansoprazole-oral-route/description/drg-20067214)

Concomitant use of multiple medications is common in clinical practice and may influence the occurrence of JIA, thereby affecting signal detection. To enhance the robustness of our analysis and minimize potential confounding bias, we conducted a sensitivity analysis by excluding reports that involved concomitant therapies. The excluded drug categories comprised nonsteroidal anti-inflammatory drugs (naproxen, diclofenac, celecoxib, ibuprofen, acetaminophen), conventional synthetic DMARDs (methotrexate, leflunomide, sulfasalazine, hydroxychloroquine, penicillamine), glucocorticoids (hydrocortisone, prednisone, dexamethasone, methylprednisolone, triamcinolone, prednisolone, budesonide, betamethasone, cortisone), biologics and targeted therapies (adalimumab, etanercept, tocilizumab, infliximab, canakinumab, anakinra, ruxolitinib, golimumab, secukinumab, tofacitinib, abatacept, sarilumab, rituximab, ustekinumab, certolizumab), and other immunosuppressants (tacrolimus, cyclosporine). After applying these exclusions, 221AE reports remained eligible for analysis, from which 16 drugs were still identified as being associated with JIA based on the criteria of four disproportionality algorithms (**Supplementary Figure S1**).

### Toxicity prediction of aripiprazole and lansoprazole

In silico toxicity predictions using ProTox-3.0 revealed multiple organ-specific toxicities associated with Lansoprazole and Aripiprazole. Computational toxicity screening of Lansoprazole using the ProTox-3.0 platform revealed a complex multi-system profile. The compound was predicted to be active for hepatotoxicity (probability = 0.53) and respiratory toxicity (0.75), with additional activity noted for blood–brain barrier permeability (0.84) and nutritional toxicity (0.80). Although nephrotoxicity and cardiotoxicity were predicted as inactive, their probabilities (0.83 and 0.72, respectively) suggest potential off-target effects in renal and cardiovascular systems (**Supplementary Table S5**). At the mechanistic level, Lansoprazole was predicted to be active toward the aryl hydrocarbon receptor (AhR) (probability = 1.00) and to interact with androgen and estrogen receptors (e.g., AR: 0.90; ER-LBD: 0.90), implying possible endocrine-disrupting properties. Additionally, the compound was flagged as active in the hERG (human Ether-à-go-go-Related Gene) channel (0.65) and cytochrome P450 inhibition was noted, particularly for CYP1A2 (0.83) and CYP3A4 (0.77)—suggesting potential for metabolic interaction and QT prolongation risk (**Supplementary Table S5**).

And, Aripiprazole exhibits a broad and high-confidence multi-system toxicity signature. The compound was predicted to be active for neurotoxicity (probability = 0.96), respiratory toxicity (0.89), and notably, immunotoxicity (0.98). It also demonstrated high probabilities for blood–brain barrier permeability (0.96) and clinical toxicity (0.90), suggesting systemic exposure and potential for CNS-related adverse events (**Supplementary Table S6**). Mechanistically, Aripiprazole was predicted to interact with several key targets relevant to immune and nervous system function. It showed strong activity toward the N-methyl-D-aspartate receptor (NMDAR; 0.94) and voltage-gated sodium channels (VGSC; 0.65)—both of which are implicated in neuroinflammation and excitotoxicity. In addition, it activated multiple cellular stress response pathways, including the heat shock element (HSE; 0.92) and ATPase-related stress signaling (0.98), indicating potential for mitochondrial and proteostatic stress. While interactions with nuclear hormone receptors (estrogen or androgen receptors) were largely inactive, metabolic pathway predictions indicated inhibition of CYP1A2 (0.83) and CYP2D6 (0.75) (**Supplementary Table S6**).

### Target intersection and network analysis

**Figure 5:**
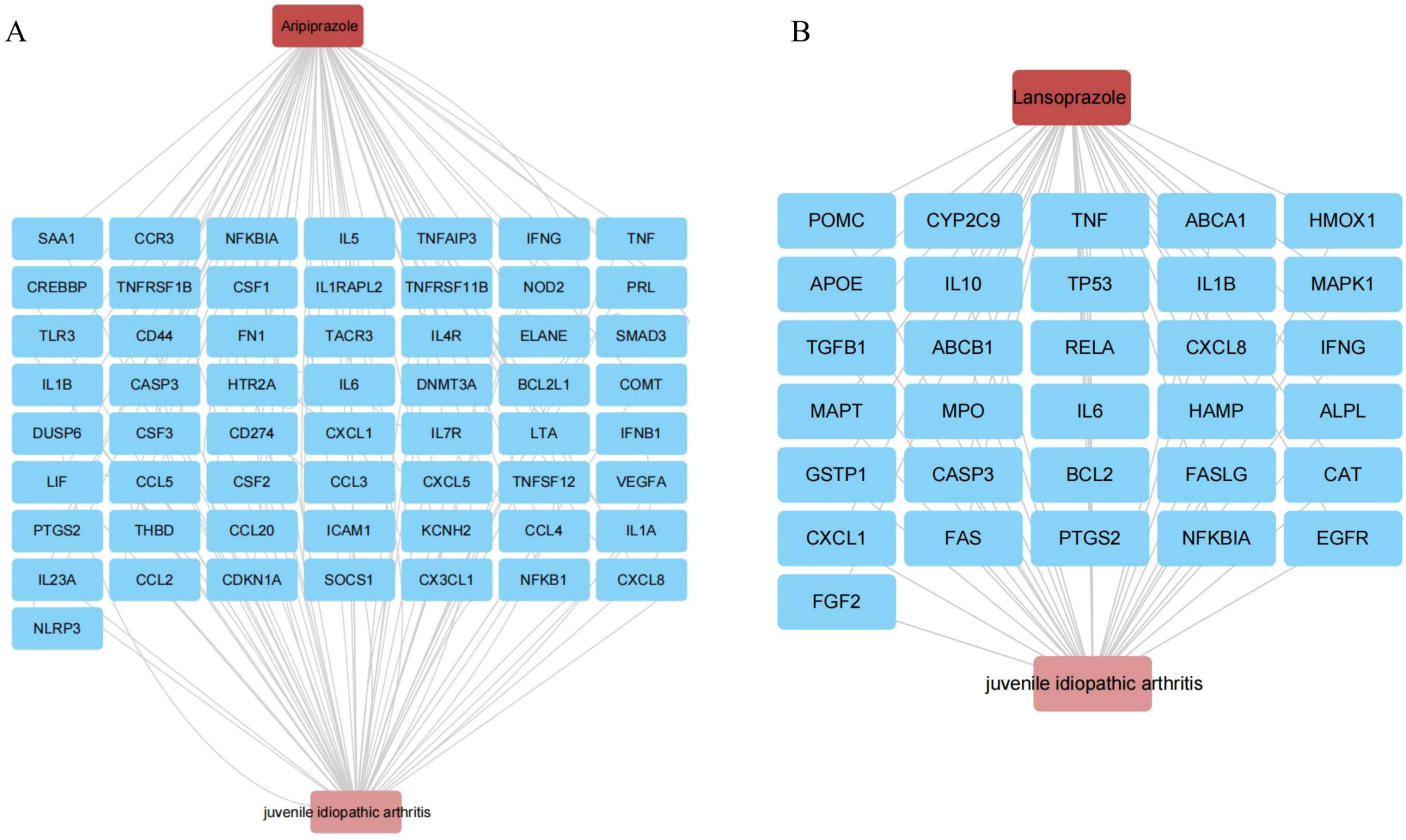
Target intersection and network analysis. **(A)** Aripiprazole and JIA interaction network in this study. **(B)** Lansoprazole and JIA interaction network in this study.

To elucidate potential mechanistic links between aripiprazole, lansoprazole and JIA, we performed a target intersection analysis. Aripiprazole and lansoprazole-associated targets were retrieved from the ChEMBL database (**Supplementary Tables S7**, **S8**), while disease-related genes were identified using GeneCards. The overlap between the two sets yielded 43 and 31 intersecting genes between aripiprazole, lansoprazole and JIA, suggesting shared molecular relevance (**Supplementary Tables S9**, **S10**). For Aripiprazole, network mapping revealed interactions with a core set of inflammation-related genes, including TNF, IL6, CXCL8, IL1B, and CASP3. These genes are known mediators of innate immune activation and cytokine signaling in JIA. Aripiprazole also intersected with TP53, RELA, and MAPK1, suggesting a potential role in modulating apoptotic and NF-κB pathways (**Figure 5A**). Similarly, Lansoprazole was linked to a highly overlapping immune-inflammation axis. Key nodes included IL6, IL1B, TNF, CXCL8, and NFKBIA, alongside regulators of oxidative stress and immune resolution such as PTGS2 and CASP3 (**Figure 5B**). These interactions support a plausible immunotoxic profile, particularly in genetically predisposed individuals.

### Association of candidate drugs with JIA subtypes

**Figure 6:**
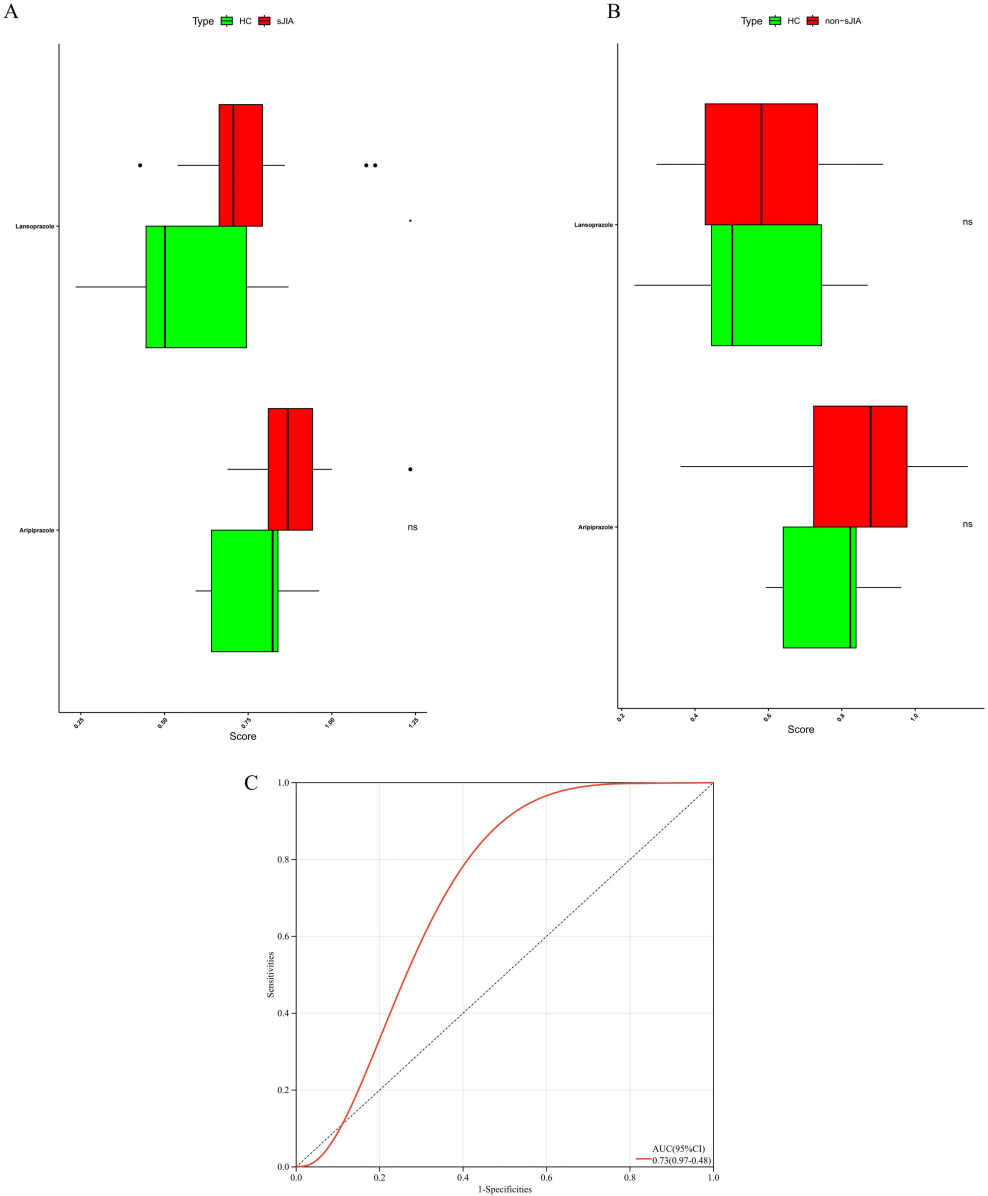
Transcriptomic activity of aripiprazole- and lansoprazole-responsive gene signatures in distinct JIA sub-types. **(A)** ssGSEA of the lansoprazole signature in sJIA versus controls. **(B)** ssGSEA of the lansoprazole signature in non-sJIA versus controls. **(C)** ROC curves for drug-induced sJIA classifiers.

To further delineate the potential associations between the identified candidate drugs and specific subtypes of JIA, we performed bulk RNA-seq analysis on a cohort of 68 patients, stratified into sJIA and non-sJIA. Using ssGSEA, we quantified drug-specific gene expression signatures. Among the compounds analyzed, lansoprazole exhibited significantly higher enrichment scores in sJIA patients compared to healthy controls (P<0.05), suggesting a potential transcriptional association with the sJIA (**Figures 6A, C**). In contrast, aripiprazole and other compounds did not show significant enrichment in either sJIA or non-sJIA patients (**Figure 6B**).

### Single-cell transcriptomic validation of drug–subtype associations

**Figure 7:**
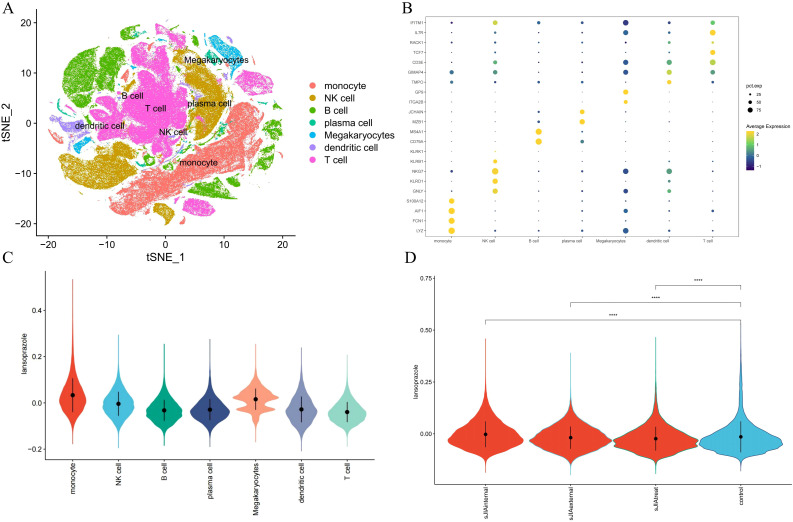
Single-cell validation of the lansoprazole–sJIA signal. **(A)** UMAP projection of PBMCs from patients and controls. **(B)** Dot plot of canonical marker genes for immune cell annotation. **(C)** Violin plots of lansoprazole signature enrichment across immune cell types. **(D)** Violin plots of signature scores in discovery/external cohorts, controls, and post-IL-6 blockade samples.

To thoroughly characterize the transcriptional landscape of sJIA patients, we conducted scRNA-seq on 27 samples from sJIA (5 centers) and 6 samples from controls, 2 paired samples from sJIA patients (n=2) before and after one month of treatment with IL-6 inhibitor**s**. After stringent quality filtering and batch effect correction of scRNA-seq data, 273671 cells were clustered into 54 clusters with an unsupervised approach, defined by well-established canonical marker genes (**Figure 7A**). These clusters were grouped into the main cellular categories: monocyte, NK cell, B cell, plasma cell, Megakaryocytes, dendritic cell, T cell (**Figure 7B**).

Cell-type–specific enrichment analysis using the AddModuleScore method revealed that lansoprazole-associated inflammatory gene signatures were preferentially enriched in monocytes and dendritic cells from sJIA patients, implicating these innate immune compartments as potential mediators of lansoprazole-driven transcriptional responses (**Figure 7C**). Furthermore, gene set scores related to lansoprazole were significantly elevated in both the internal and external sJIA cohorts, as well as in patients following IL-6 blockade therapy, compared to controls (**Figure 7D**). These single-cell–level findings reinforce the hypothesis that lansoprazole may influence disease-relevant immune pathways in sJIA, particularly through effects on monocytes activation.

**Figure 8:**
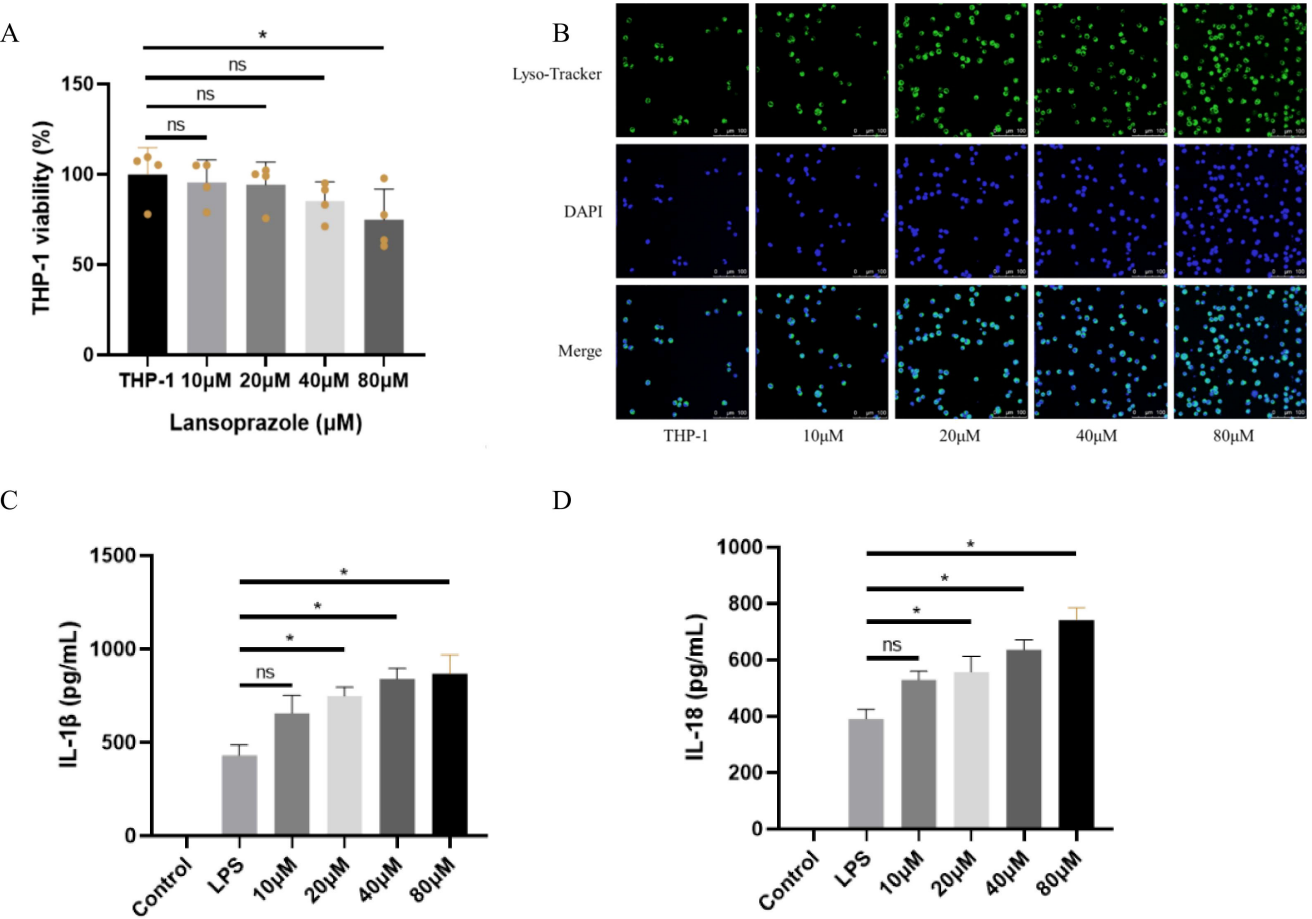
Cell-based validation of lansoprazole-induced inflammasome activation in THP-1 cell. **(A)** Cell viability measured by CCK-8 after 24h exposure to increasing concentrations of lansoprazole (0–80 μM). Lansoprazole at 80 μM significantly reduced viability, whereas ≤40 μM was non-cytotoxic. **(B)** Representative confocal images of THP-1 stained with LysoTracker (green) and DAPI (blue). Lansoprazole enhanced lysosomal activity in a concentration-dependent manner. Scale bar: 20 μm. **(C-D)**, ELISA quantification of IL-1β **(C)** and IL-18 **(D)** secretion in LPS-primed THP-1 macrophages following lansoprazole treatment. Lansoprazole significantly increased cytokine release at 20–80 μM compared with LPS alone. Data are shown as mean ± SEM. *P < 0.05; ns, not significant.

To experimentally validate the predicted mechanism, we performed cell-based assays in THP-1. CCK-8 analysis showed that lansoprazole at 80 μM significantly reduced cell viability compared with untreated controls, whereas 10–40 μM had no significant cytotoxic effect (**Figure 8A**). LysoTracker confocal imaging demonstrated a concentration-dependent increase in lysosomal activity after 24h exposure to lansoprazole, with progressively stronger fluorescence signals observed from 10 to 80 μM (**Figure 8B**). To assess inflammasome activation, cells were primed with LPS to induce baseline expression of pro-IL-1β and NLRP3, followed by lansoprazole treatment. ELISA quantification revealed that lansoprazole markedly enhanced IL-1β and IL-18 secretion in a dose-dependent manner, with significant increases detected at 20, 40, and 80 μM compared to LPS alone (**Figures 8C–D**). These findings provide functional support that lansoprazole promotes lysosomal stress and inflammasome-driven cytokine release.

## Discussion

In this study we combined large-scale pharmacovigilance mining, machine learning, and multi-center transcriptomic validation to uncover drugs that may precipitate or exacerbate juvenile idiopathic arthritis. In FAERS reports, four disproportionality algorithms converged on 40 candidate drugs; three orthogonal prediction models (DMPNN, GCN and SVM) further refined this list to 22 high-confidence compounds. Of these, lansoprazole and aripiprazole received the strongest and most consistent signals. Toxicogenomic predictions implicated both drugs in innate-immune and stress-response pathways, and lansoprazole-associated signatures were selectively enriched in systemic JIA (sJIA) bulk RNA-seq samples as well as monocyte- and dendritic-cell clusters from single-cell datasets. Collectively, these multi-tier data identify lansoprazole as a plausible pharmacological trigger in sJIA and illustrate the utility of AI-driven frameworks for drug safety surveillance in pediatric rheumatology.

Lansoprazole is a proton-pump inhibitor (PPI) that has been widely prescribed since its approval in 1995 for gastro-esophageal reflux disease, peptic-ulcer disease, Helicobacter pylori eradication regimens and Zollinger-Ellison syndrome (28, 29). After protonation in the acidic gastric milieu, the pro-drug is converted to an active sulfenamide that covalently binds the parietal-cell H^+^/K^+^-ATPase, thereby blocking the final step of gastric acid secretion (30). Following oral administration, the drug reaches Cmax at approximately and displays 80–85% bioavailability; it is metabolized mainly by CYP2C19 and CYP3A4. Although its plasma half-life is only ~1.5 h, acid suppression persists for ≥ 24h. Common adverse effects include headache, abdominal bloating, diarrhea or constipation, and rash; long-term or high-dose therapy has been linked to hypomagnesaemia, vitamin-B_12_ deficiency, fractures and Clostridioides difficile infection (31, 32). Isolated case reports further implicate PPIs—including lansoprazole—in DILE, whose hallmark triad of persistent fever, malar rash and arthralgia/arthritis closely mirrors the febrile and arthritic phenotype of sJIA (1, 33). Our multi-modal analyses—comprising FAERS pharmacovigilance signals, high-risk scores from AI prediction models, and significant enrichment of lansoprazole gene signatures in sJIA monocyte and dendritic-cell populations in both bulk and single-cell RNA-seq—provide convergent evidence that lansoprazole may trigger or exacerbate sJIA-like inflammatory responses in predisposed children. Clinicians should therefore weigh benefits against potential risks when prescribing lansoprazole to patients with underlying autoimmune tendencies or those requiring long-term PPI therapy, monitor electrolytes, bone density and inflammatory markers, and reassess treatment if rashes or joint symptoms arise.

Although the molecular connection between lansoprazole and the pathogenesis of sJIA has yet to be fully defined, accumulating evidence indicates that this drug can disrupt innate-immune homeostasis through multiple convergent routes and precipitate an amplified inflammatory cascade. First, in human myeloid cells the drug inhibits lysosomal V-ATPase activity by ~70%, thereby alkalinizing autophago-lysosomal compartments, blocking cargo degradation and licensing cathepsin-mediated NLRP3-inflammasome assembly and IL-1β/IL-18 maturation. Concomitantly, the same lysosomal stress triggers PERK, IRE1 and ATF6 activation, launching a CHOP-dominated unfolded-protein response that further amplifies pro-inflammatory cytokine release (34). Second, chronic PPI therapy precipitates systemic hypomagnesaemia; murine studies show that Mg^2+^ depletion alone primes monocytes and dendritic cells for exaggerated NLRP3 activation and elevates circulating IL-1β, identifying electrolyte imbalance as an independent feed-forward amplifier of inflammasome signaling (35, 36). Third, sustained acid suppression reshapes the gut microbiota towards Gram-negative, LPS-rich taxa, a dysbiosis epidemiologically linked to Clostridioides difficile infection and experimentally associated with higher systemic IL-6 and IL-1 family cytokines (37). Finally, these lysosomal, ER-stress, electrolyte and microbiome insults converge on an NLRP3-dependent IL-1/IL-18 surge coupled to IL-6/STAT3 trans-activation—the canonical cytokine axis driving systemic JIA flares—thus providing a coherent molecular rationale for our multi-omics evidence that implicates lansoprazole as a plausible pharmacological trigger of sJIA-like inflammation in genetically susceptible children.

In addition to pharmacovigilance and multi-omics evidence, our cell-based experiments provide direct molecular support for the proposed mechanism. Using THP-1 cell, we confirmed that non-cytotoxic concentrations of lansoprazole enhanced lysosomal activity and significantly increased IL-1β and IL-18 secretion in LPS-primed cells. These findings are consistent with a model in which lansoprazole perturbs lysosomal V-ATPase function, induces lysosomal stress, and thereby licenses NLRP3 inflammasome activation. Importantly, the observed cytokine release occurred only after LPS priming, highlighting lansoprazole as a secondary trigger that amplifies pre-existing inflammatory signals. Taken together with FAERS disproportionality signals, AI-based predictions, and transcriptomic enrichment in sJIA monocytes, the wet-lab validation strengthens the link between lansoprazole exposure and inflammasome-driven inflammation. While further studies are required to directly assess V-ATPase activity and NLRP3/caspase-1 cleavage in primary patient samples, our integrated framework provides convergent evidence that lansoprazole may act as a pharmacological trigger in sJIA.

Nevertheless, our current validation was restricted to THP-1, which represent the most relevant immune cell model for sJIA pathogenesis. Broader applicability should be assessed in diverse cellular systems, such as hepatoma lines (HepG2.2.15, Huh7) and normal liver cells (LO2), which have been widely used to evaluate drug-induced immunotoxicity and hepatotoxicity (38).

In summary, Given the widespread use of PPIs in children for gastro-esophageal reflux or steroid-induced gastritis, our data suggest that lansoprazole should be prescribed judiciously in patients with, or at risk for, sJIA. Prospective monitoring of musculoskeletal symptoms and inflammatory markers after PPI initiation may help clarify temporal relationships. Integration of AI-based screening into routine pharmacovigilance pipelines could enable earlier detection of similar subtype-specific safety signals, informing shared decision-making between clinicians and families.

Our work leverages three major strengths: (i) comprehensiveness, drawing on FAERS records; (ii) methodological triangulation, with concordant predictions from descriptor-based ML and graph-based DL; and (iii) external validation across bulk and single-cell cohorts from five centers. Nevertheless, several limitations warrant caution. First, FAERS is subject to under-reporting, indication bias and confounding by co-medication; disproportionality signals do not prove causality. Second, the RNA-seq cohorts were modest in size and lacked direct drug-exposure information; consequently, ssGSEA and AddModuleScore analyses capture transcriptional convergence compatible with drug signatures rather than confirming actual intake. Future studies should integrate real-world prescription records with paired transcriptomic datasets. Finally, toxicity predictions were supported by complementary cell-based assays, but further validation in primary immune cells and detailed molecular experiments will be required to fully elucidate the underlying mechanisms.

By integrating pharmacovigilance analytics, machine-learning prediction, and multi-layer transcriptomics, we provide convergent evidence that lansoprazole is preferentially linked to sJIA and outline a generalizable roadmap for uncovering hidden drug–disease interactions in rare pediatric disorders. These insights highlight the importance of cross-disciplinary data integration for safeguarding vulnerable patient populations while optimizing therapeutic outcomes.

## Data Availability

The datasets presented in this study can be found in online repositories. The names of the repository/repositories and accession number(s) can be found in the article/**Supplementary Material**.

## Supplementary material

Supplementary Figure 1 Sensitivity analysis of drug–JIA associations after excluding concomitant therapies.

## References

[1] Jacobson JL Pham JT. Juvenile idiopathic arthritis: A focus on pharmacologic management. J Pediatr Health Care. (2018) 32:515–28. doi: 10.1016/j.pedhc.2018.02.005, PMID: 30177013

[2] Guzman J Oen K Huber AM Watanabe Duffy K Boire G Shiff N. The risk and nature of flares in juvenile idiopathic arthritis: results from the ReACCh-Out cohort. Ann Rheum Dis. (2016) 75:1092–8. doi: 10.1136/annrheumdis-2014-207164, PMID: 25985972

[3] Luo Q Hao H Xiwen L Qiu X Liu D Liu Y. UBE2D1 as a key biomarker in systemic juvenile idiopathic arthritis: a new perspective on diagnosis and disease activity assessment. Arthritis Res Ther. (2025) 27:140. doi: 10.1186/s13075-025-03606-8, PMID: 40635084 PMC12239252

[4] Horton DB Scott FI Haynes K Putt ME Rose CD Lewis JD. Antibiotic exposure and juvenile idiopathic arthritis: A case-control study. Pediatrics. (2015) 136:e333–43. doi: 10.1542/peds.2015-0036, PMID: 26195533 PMC4516942

[5] Clarke SLN Mageean KS Maccora I Arrison S Simonini G Sharp GC. Moving from nature to nurture: a systematic review and meta-analysis of environmental factors associated with juvenile idiopathic arthritis. Rheumatol (Oxford). (2022) 61:514–30. doi: 10.1093/rheumatology/keab627, PMID: 34382060 PMC8824412

[6] Ronaghy A de Jager W Zonneveld-Huijssoon E Klein MR van Wijk F Rijkers GT. Vaccination leads to an aberrant FOXP3 T-cell response in non-remitting juvenile idiopathic arthritis. Ann Rheum Dis. (2011) 70:2037–43. doi: 10.1136/ard.2010.145151, PMID: 21859687

[7] Larivuo I Laukkala H Nevalainen A Arponen O Nevalainen OPO. Psychiatric medications and the risk of autoimmune and immune-mediated inflammatory diseases: A systematic review and meta-analysis of observational studies. PLoS One. (2023) 18(2):e0281979. doi: 10.1371/journal.pone.0281979, PMID: 36854031 PMC9974122

[8] Liu J Wu H Xia SL. New-onset arthritis following COVID-19 vaccination: A systematic review of case reports. Vaccines (Basel). (2023) 11:665. doi: 10.3390/vaccines11030665, PMID: 36992249 PMC10055862

[9] LeBaron CW Bi D Sullivan BJ Beck C Gargiullo P. Evaluation of potentially common adverse events associated with the first and second doses of measles-mumps-rubella vaccine. Pediatrics. (2006) 118:1422–30. doi: 10.1542/peds.2006-0678, PMID: 17015532

[10] Shimizu M Ueno K Yachie A. Relapse of systemic juvenile idiopathic arthritis after influenza vaccination in a patient receiving tocilizumab. Clin Vaccine Immunol. (2012) 19:1700–2. doi: 10.1128/CVI.00309-12, PMID: 22875602 PMC3485886

[11] Bellucca S Calvo PL Giugliano L Opramolla A. A case of paradoxical arthralgia following anti-TNF monoclonal antibody administration in a patient with new-onset pediatric Crohn's disease. JPGN Rep. (2023) 4:e308. doi: 10.1097/PG9.0000000000000308, PMID: 37200710 PMC10187859

[12] Alivernini S Pugliese D Tolusso B Bui L Petricca L Guidi L. Paradoxical arthritis occurring during anti-TNF in patients with inflammatory bowel disease: histological and immunological features of a complex synovitis. RMD Open. (2018) 4:e000667. doi: 10.1136/rmdopen-2018-000667, PMID: 29657833 PMC5892785

[13] Solhjoo M Goyal A Chauhan K. Drug-induced lupus erythematosus. In: StatPearls. StatPearls Publishing, Treasure Island (FL (2023)., PMID: 28722919

[14] Ponce A Frade-Sosa B Sarmiento-Monroy JC Sapena N Ramírez J Azuaga AB. Imaging findings in patients with immune checkpoint inhibitor-induced arthritis. Diagnostics (Basel). (2022) 12:1961. doi: 10.3390/diagnostics12081961, PMID: 36010310 PMC9406920

[15] Kim ST Chu Y Misoi M Suarez-Almazor ME Tayar JH Lu H. Distinct molecular and immune hallmarks of inflammatory arthritis induced by immune checkpoint inhibitors for cancer therapy. Nat Commun. (2022) 13:1970. doi: 10.1038/s41467-022-29539-3, PMID: 35413951 PMC9005525

[16] Kindgren E Ludvigsson J. Infections and antibiotics during fetal life and childhood and their relationship to juvenile idiopathic arthritis: a prospective cohort study. Pediatr Rheumatol Online J. (2021) 19:145. doi: 10.1186/s12969-021-00611-4, PMID: 34530851 PMC8447683

[17] Ornetti P Disson-Dautriche A Muller G Cherasse A Tavernier C Besancenot JF. Joint symptoms in patients on bupropion therapy. Joint Bone Spine. (2004) 71:583–5. doi: 10.1016/j.jbspin.2003.10.004, PMID: 15589445

[18] Yu Z Kou F Gao Y Gao F Lyu CM Wei H. A machine learning model for predicting abnormal liver function induced by a Chinese herbal medicine preparation (Zhengqing Fengtongning) in patients with rheumatoid arthritis based on real-world study. J Integr Med. (2025) 23:25–35. doi: 10.1016/j.joim.2024.12.001, PMID: 39721810

[19] Ye W Yu Y Zhu X Wan W Liu Y Zou H. A Common Functional Variant at the Enhancer of the Rheumatoid Arthritis Risk Gene *ORMDL3* Regulates its Expression Through Allele-Specific JunD Binding. Phenomics. (2023) 3:485–95. doi: 10.1007/s43657-023-00107-z, PMID: 37881318 PMC10593690

[20] Raschi E Antonazzo IC La Placa M Ardizzoni A Poluzzi E De Ponti F. Serious cutaneous toxicities with immune checkpoint inhibitors in the U.S. Food and drug administration adverse event reporting system. Oncologist. (2019) 24:e1228–31. doi: 10.1634/theoncologist.2019-0250, PMID: 31387950 PMC6853099

[21] Ramos-Tiñini P Menchaca-Aguayo H Alpizar-Rodriguez D Mercedes-Pérez E Faugier-Fuentes E. Application of the new classification proposal for juvenile idiopathic arthritis of the pediatric rheumatology international trials organization in a group of Mexican patients. Front Pediatr. (2024) 12:1476257. doi: 10.3389/fped.2024.1476257, PMID: 39575112 PMC11578732

[22] Tian X Chen L Gai D He S Jiang X Zhang N. Adverse event profiles of PARP inhibitors: analysis of spontaneous reports submitted to FAERS. Front Pharmacol. (2022) 13:851246. doi: 10.3389/fphar.2022.851246, PMID: 35401230 PMC8990839

[23] Sato K Niimi Y Mano T Iwata A Iwatsubo T. Time to onset of drug-induced parkinsonism: Analysis using a large Japanese adverse event self-reporting database. Biosci Trends. (2022) 16:151–7. doi: 10.5582/bst.2022.01115, PMID: 35444114

[24] Zhao Z Luo Q Liu Y Jiang K Zhou L Dai R. Multi-level integrative analysis of the roles of lncRNAs and differential mRNAs in the progression of chronic pancreatitis to pancreatic ductal adenocarcinoma. BMC Genomics. (2023) 24:101. doi: 10.1186/s12864-023-09209-4, PMID: 36879212 PMC9990329

[25] Ji Q Zheng Y Zhang G Hu Y Fan X Hou Y. Single-cell RNA-seq analysis reveals the progression of human osteoarthritis. Ann Rheum Dis. (2019) 78:100–10. doi: 10.1136/annrheumdis-2017-212863, PMID: 30026257 PMC6317448

[26] Choudhary S Satija R. Comparison and evaluation of statistical error models for scRNA-seq. Genome Biol. (2022) 23:27. doi: 10.1186/s13059-021-02584-9, PMID: 35042561 PMC8764781

[27] KKorsunsky I Millard N Fan J Slowikowski K Zhang F Wei K. Fast, sensitive and accurate integration of single-cell data with Harmony. Nat Methods. (2019) 16:1289–96. doi: 10.1038/s41592-019-0619-0, PMID: 31740819 PMC6884693

[28] Matheson AJ Jarvis B. Lansoprazole: an update of its place in the management of acid-related disorders. Drugs. (2001) 61:1801–33. doi: 10.2165/00003495-200161120-00011, PMID: 11693467

[29] Raimi-Abraham BT Garcia Del Valle A Varon Galcera C Barker SA Orlu M. Investigating the physical stability of repackaged medicines stored into commercially available multicompartment compliance aids (MCAs). J Pharm Health Serv Res. (2017) 8:81–9. doi: 10.1111/jphs.12176, PMID: 28713440 PMC5488225

[30] Marker T Steimbach RR Perez-Borrajero C Luzarowski M Hartmann E Schleich S. Site-specific activation of the proton pump inhibitor rabeprazole by tetrathiolate zinc centres. Nat Chem. (2025) 17:507–17. doi: 10.1038/s41557-025-01745-8, PMID: 39979415 PMC11964933

[31] Lam JR Schneider JL Zhao W Corley DA. Proton pump inhibitor and histamine 2 receptor antagonist use and vitamin B12 deficiency. JAMA. (2013) 310:2435–42. doi: 10.1001/jama.2013.280490, PMID: 24327038

[32] Yibirin M De Oliveira D Valera R Plitt AE Lutgen S. Adverse effects associated with proton pump inhibitor use. Cureus. (2021) 13:e12759. doi: 10.7759/cureus.12759, PMID: 33614352 PMC7887997

[33] Bataille P Lebrun-Vignes B Tubach F Aroux-Pavard M Philibert C Chasset F. Proton pump inhibitors associated with drug-induced lupus erythematosus. JAMA Dermatol. (2022) 158:1208–10. doi: 10.1001/jamadermatol.2022.2421, PMID: 35976639 PMC9386607

[34] Lee WP. Suppression of vacuolar-type ATPase and induction of endoplasmic reticulum stress by proton pump inhibitors. J Chin Med Assoc. (2022) 85:915–21. doi: 10.1097/JCMA.0000000000000785, PMID: 36150104 PMC12755635

[35] Zhao W Zhang J Jia H He Q Cui J Ding L. Proton pump inhibitor-induced hypomagnesemia, a rare cause of reversible delirium: A case report with literature review. Med (Baltimore). (2024) 103:e39729. doi: 10.1097/MD.0000000000039729, PMID: 39465769 PMC11460912

[36] Pitzer Mutchler A Huynh L Patel R Lam T Bain D Jamison S. The role of dietary magnesium deficiency in inflammatory hypertension. Front Physiol. (2023) 14:1167904. doi: 10.3389/fphys.2023.1167904, PMID: 37293263 PMC10244581

[37] Kiecka A Szczepanik M. Proton pump inhibitor-induced gut dysbiosis and immunomodulation: current knowledge and potential restoration by probiotics. Pharmacol Rep. (2023) 75:791–804. doi: 10.1007/s43440-023-00489-x, PMID: 37142877 PMC10159235

[38] Mirzaei R Afaghi A Babakhani S Sohrabi MR Hosseini-Fard SR Babolhavaeji K. Role of microbiota-derived short-chain fatty acids in cancer development and prevention. BioMed Pharmacother. (2021) 139:111619. doi: 10.1016/j.biopha.2021.111619, PMID: 33906079

