# Supplementary figures and images for: Integrative pharmacovigilance and AI-based framework uncovers potential drug triggers in juvenile idiopathic arthritis
Source: Front Immunol. 2025 Nov 3;16:1653003. doi: 10.3389/fimmu.2025.1653003 (PMC12620426; doi:10.3389/fimmu.2025.1653003)

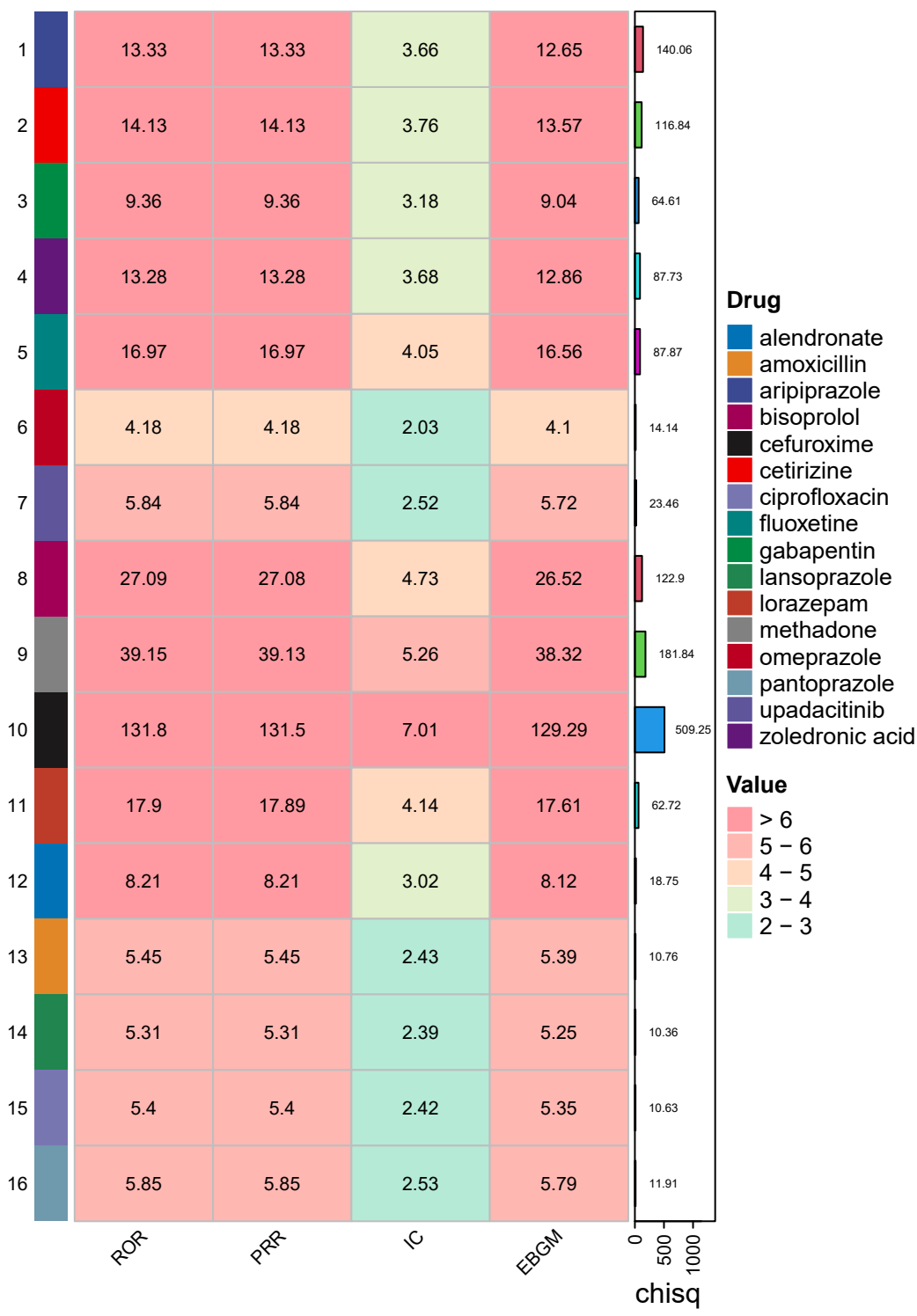

Supplement: Supplementary Figure 1 — Sensitivity analysis of drug–JIA associations after excluding concomitant therapies. [file Image1.pdf]
